# Clinical Characteristics and Outcomes of Patients with Lung Cancer and Venous Thromboembolism

**DOI:** 10.1055/s-0038-1656542

**Published:** 2018-06-01

**Authors:** Pedro Ruiz-Artacho, Javier Trujillo-Santos, Luciano López-Jiménez, Carme Font, María del Carmen Díaz-Pedroche, Juan Francisco Sánchez Muñoz-Torrero, Maria Luisa Peris, Andris Skride, Ana Maestre, Manuel Monreal, the RIETE Investigators

**Affiliations:** 1 Department of Emergency, Hospital Clínico San Carlos, Madrid, Spain; 2 Department of Internal Medicine, Hospital General Universitario Santa Lucía, Murcia, Spain; 3 Department of Internal Medicine, Hospital Universitario Reina Sofía, Córdoba, Spain; 4 Department of Medical Oncology, Hospital Clínic, Barcelona, Spain; 5 Department of Internal Medicine, Hospital Universitario 12 de Octubre, Madrid, Spain; 6 Department of Internal Medicine, Hospital San Pedro de Alcántara, Cáceres, Spain; 7 Department of Internal Medicine, Consorcio Hospitalario Provincial de Castellón, CEU Cardenal Herrera University, Castellón, Spain; 8 Department of Cardiology, Pauls Stradins Clinical University Hospital, Riga, Latvia; 9 Department of Internal Medicine, Hospital Universitario de Vinalopó, Elche, Alicante, Spain; 10 Department of Internal Medicine, Hospital Germans Trias i Pujol, Universidad Autónoma de Barcelona, Badalona, Barcelona, Spain

**Keywords:** venous thrombosis, pulmonary embolism, recurrences, bleeding, anticoagulant therapy, lung cancer

## Abstract

**Background** The natural history of patients with lung cancer and venous thromboembolism (VTE) has not been consistently evaluated.

**Methods** We used the RIETE (Registro Informatizado Enfermedad TromboEmbólica) database to assess the clinical characteristics, time course, and outcomes during anticoagulation of lung cancer patients with acute, symptomatic VTE.

**Results** As of May 2017, a total of 1,725 patients were recruited: 1,208 (70%) presented with pulmonary embolism (PE) and 517 with deep vein thrombosis (DVT). Overall, 865 patients (50%) were diagnosed with cancer <3 months before, 1,270 (74%) had metastases, and 1,250 (72%) had no additional risk factors for VTE. During anticoagulation (median, 93 days), 166 patients had symptomatic VTE recurrences (recurrent DVT: 86, PE: 80), 63 had major bleeding (intracranial 11), and 870 died. The recurrence rate was twofold higher than the major bleeding rate during the first month, and over threefold higher beyond the first month. Fifty-seven patients died of PE and 15 died of bleeding. Most fatal PEs (84%) and most fatal bleeds (67%) occurred within the first month of therapy. Nine patients with fatal PE (16%) died within the first 24 hours. Of 72 patients dying of PE or bleeding, 15 (21%) had no metastases and 29 (40%) had the VTE shortly after surgery or immobility.

**Conclusion** Active surveillance on early signs and/or symptoms of VTE in patients with recently diagnosed lung cancer and prescription of prophylaxis in those undergoing surgery or during periods of immobilization might likely help prevent VTE better, detect it earlier, and treat it more efficiently.

## Introduction

Lung cancer is the most common malignancy, and a leading cause of death. 1 Patients with lung cancer are at increased risk for venous thromboembolism (VTE), 2 3 4 5 6 and VTE appearing in patients with cancer carries an increased risk for early mortality. 2 7 Unfortunately, however, there is scarce information in the literature on the clinical presentation, time-course, and clinical outcomes of lung cancer patients developing VTE in real-life practice. A better knowledge of the burden of VTE in lung cancer patients, linked to adequate dissemination of the data, could likely help improve the awareness of attending doctors, nurses, and patients. A better understanding of at-risk patients and time course of VTE could likely help design better prevention strategies and detect VTE earlier. Finally, a better information on the natural history of VTE during the course of anticoagulant therapy could likely help design better therapeutic strategies (drugs, doses, duration) for this subgroup of patients with cancer.

The RIETE ( *R* egistro *I* nformatizado *E* nfermedad *T* rombo *E* mbólica) registry is an ongoing, multicenter, international, observational registry of consecutive patients with objectively confirmed acute VTE (ClinicalTrials.gov identifier: NCT02832245). Data from this registry have been used to evaluate outcomes after acute VTE, such as the frequency of recurrent VTE, bleeding and mortality, and risk factors for these outcomes. 8 9 10 11 12 The aim of the current study was to assess the clinical characteristics, time course of VTE, and clinical outcomes during anticoagulant therapy in all patients with lung cancer presenting with symptomatic VTE.

## Methods

This study is an analysis of prospectively collected data in the RIETE registry, from 209 hospitals in Europe, America, and Asia. RIETE included consecutive patients with deep vein thrombosis (DVT) or pulmonary embolism (PE) confirmed by objective tests (compression ultrasonography or contrast venography for suspected DVT; pulmonary angiography, ventilation–perfusion lung scan, or helical computed tomography [CT] scan for suspected PE) since March 2001. Its design, rationale, and methodology have been reported elsewhere. 13 Exclusion criteria were a current enrollment in a therapeutic clinical trial with a blinded therapy. Informed consent was obtained from all participants in accordance with local ethics committee requirements.

All patients with biopsy-proven active cancer in the lung presenting with acute symptomatic, objectively confirmed VTE were considered for this study. Those with incidentally found VTE were excluded. Characteristics of the index VTE episode and of patients (demographics, comorbidities, additional risk factors for VTE, concomitant medications, initial and long-term therapy, and outcomes during anticoagulation) were recorded. VTE was considered to be secondary to surgery if appearing within 2 months of the procedure, and secondary to immobilization if within 2 months of confinement to bed with bathroom privileges for ≥4 days. Active cancer included cancer diagnosed within the 3 months prior to the incident VTE, metastatic cancer, or cancer with current therapy (surgery, chemotherapy, radiotherapy, hormonal or support therapy).

Patients were managed according to the clinical practice of each participating hospital (i.e., there was no standardization of treatment). The type, dose, and duration of anticoagulant therapy were recorded. After VTE diagnosis, all patients were followed up in the outpatient clinic for at least 3 months. During each visit, any signs or symptoms suggesting recurrent DVT or PE or major bleeding were noted. All episodes of clinically suspected VTE recurrences were investigated by repeat compression ultrasonography, helical CT pulmonary, ventilation–perfusion lung scintigraphy, angiography, or pulmonary angiography. Recurrent VTE was defined as a DVT in a new segment, a DVT 4 mm larger in diameter when compared with prior venous ultrasound, a new ventilation–perfusion mismatch in a lung scan, or a new intraluminal filling defect on a CT scan. Bleeding complications were classified as “major” if they were overt and required a transfusion of two units of blood or more; or were retroperitoneal, spinal, or intracranial; or when they were fatal. This definition is similar to that from the ISTH, with the only exception that in RIETE we do not consider a decrease of 2 g/dL of hemoglobin as a criterion **.** Fatal PE, in the absence of autopsy, was defined as any death appearing within 10 days after symptomatic PE diagnosis, in the absence of any alternative cause of death. Fatal bleeding was considered as any death appearing within 10 days after a major bleed, in the absence of any alternative cause of death.

Categorical variables were compared using the chi-square test (two-sided) and Fisher's exact test (two-sided). Continuous variables were compared using Student's *t* -test. Hazard ratios (HR) and corresponding 95% confidence intervals (CIs) were calculated. Incidence rates were calculated as cumulative incidence (events/100 patient-years) and compared using the HRs. All analyses used time-to-event methods. Risks of recurrent VTE or major bleeding were assessed with proportional hazard Cox models. Time zero was the date of diagnosis of VTE and patients were censored at the time of discontinuation of anticoagulation, at the time of death, or at the last date for which outcome data were available. The assumption of independence of the time distribution between outcomes (VTE recurrences or major bleeding) and death may not be satisfied in survival analysis due to the competing risk of death. Therefore, the Kaplan–Meier method was not appropriate to estimate survival curves for VTE. Kaplan–Meier method and log-rank test were used to compare the function of cumulative incidence for each outcome between both groups of patients (PE patients group vs. DVT patients group). Then a competing risk analysis with the Fine and Gray method was performed. Covariates included in the adjusted model were those for which a statistically significant difference (a threshold *p* -value of 0.1 was set to assess significance of differences) was found between the different variables, and a backward selection was used for the covariate selection in the multivariable model. Statistical analyses were conducted with SPSS for Windows Release (version 20; SPSS Inc. Chicago, Illinois, United States).

## Results

As of May 2017, a total of 10,962 patients with active cancer were recruited in RIETE, of whom 1,725 (16%) had lung cancer. There were 1,250 men (72%) and 475 women, aged 65 ± 12 years, and most (74%) had metastases. Overall, 1,208 patients (70%) presented with symptomatic PE (with or without concomitant DVT) and 517 with symptomatic DVT alone.

VTE was diagnosed during the first 90 days after cancer diagnosis in 865 patients (50%), including 546 (32%) in whom VTE occurred during the first 30 days ( Table 1 ). Additional risk factors for VTE included recent surgery (7.5%) or immobilization (20%). Eighty-four of 129 patients with recent surgery (65%) and 111 of 346 with recent immobilization (32%) had received VTE prophylaxis. Half of the patients (51%) were receiving chemotherapy at baseline, and 23% were receiving corticosteroids. Among 1,208 patients presenting with PE, 1,051 (87%) had dyspnea, 462 (38%) chest pain, 95 (7.9%) hemoptysis, and 94 (7.8%) had syncope. Among 517 patients presenting with DVT, 124 (24%) had upper extremity DVT (secondary to catheter in 51). At VTE diagnosis, 1,007 patients (58%) had anemia, 359 (21%) had a platelet count lower than 100,000/µL, and 468 (27%) had creatinine clearance (CrCl) levels lower than 60 mL/min.

**Table 1:** Clinical characteristics of the patients, according to initial VTE presentation

	Pulmonary embolism	Deep vein thrombosis	Odds ratio (95% CI)
**Patients** , ***N***	**1,208**	**517**	
Clinical characteristics			
Male gender	873 (72%)	377 (73%)	0.97 (0.77–1.22)
Age (mean y ± SD)	65 ± 12	65 ± 12	–
Body weight (mean kg ± SD)	72 ± 14	72 ± 14	–
Cancer characteristics			
Metastatic	883 (73%)	387 (75%)	0.91 (0.72–1.16)
Time elapsed since cancer diagnosis			
< 90 d	607 (50%)	258 (50%)	1.01 (0.83–1.25)
< 30 d	399 (33%)	147 (28%)	1.24 (0.99–1.56)
Cancer therapy			
Radiotherapy	50 (4.1%)	28 (5.4%)	0.75 (0.47–1.21)
Chemotherapy	468 (39%)	198 (38%)	1.02 (0.82–1.26)
Radio- and chemotherapy	151 (13%)	63 (12%)	1.03 (0.75–1.41)
Underlying conditions			
Chronic lung disease	72 (6.0%)	19 (3.7%)	1.66 (0.99–2.78)
Chronic heart failure	316 (26%)	99 (19%)	1.50 (1.16–1.93)
Additional risk factors for VTE			
Recent surgery	104 (8.6%)	25 (4.8%)	1.85 (1.18–2.91)
VTE prophylaxis (yes)	67 (64%)	17 (68%)	0.85 (0.32–2.16)
Immobility ≥4 d	243 (20%)	103 (20%)	1.01 (0.78–1.31)
VTE prophylaxis (yes)	78 (32%)	33 (32%)	1.00 (0.61–1.66)
Use of estrogens	14 (1.2%)	4 (0.8%)	1.50 (0.49–4.59)
None of the above	871 (72%)	391 (76%)	0.83 (0.66–1.06)
Prior VTE	123 (10%)	66 (13%)	0.78 (0.56–1.07)
Concomitant therapies			
Antiplatelets	159 (13%)	76 (15%)	0.88 (0.66–1.18)
Corticosteroids	282 (23%)	112 (22%)	1.10 (0.86–1.41)
Blood tests			
Anemia	678 (56%)	329 (64%)	0.73 (0.59–0.90)
Platelet count <100,000/µL	245 (20%)	114 (22%)	0.90 (0.70–1.15)
Platelet count >450,000/µL	90 (7.5%)	25 (4.8%)	1.58 (0.99–2.49)
CrCl levels <60 mL/min	322 (27%)	146 (28%)	0.92 (0.73–1.16)

Abbreviations: CI, confidence intervals; CrCl, creatinine clearance; SD, standard deviation; VTE, venous thromboembolism.

Most patients (90%) were initially treated with low-molecular-weight heparin (LMWH), independently of initial presentation as PE or DVT, but those with PE were more likely to receive unfractionated heparin (UFH) or thrombolytics than those with DVT alone ( Table 2 ). Then, most patients in both subgroups (65%) continued on LMWH for long-term therapy. A small proportion of patients received Fondaparinux (1.4%), direct oral anticoagulants (0.5%), or a vena cava filter (1.7%).

**Table 2:** Treatment strategies, according to initial VTE presentation

	Pulmonary embolism	Deep vein thrombosis	Odds ratio (95% CI)
**Patients** , ***N***	**1,208**	**517**	
Duration of therapy			
Mean days ( ± SD)	124 ± 140	149 ± 247	*p* = 0.008
Median days (interquartile range)	93 (125)	92 (155)	*p* = 0.80
Duration >6 months	307 (26%)	106 (21%)	1.32 (1.03–1.69)
Initial therapy			
LMWH	1,067 (88%)	478 (93%)	0.62 (0.43–0.89)
Mean LMWH dose (IU/kg/d)	177 ± 42	175 ± 48	*p* = 0.39
Unfractionated heparin	93 (7.7%)	19 (3.7%)	2.19 (1.32–3.62)
Fondaparinux	18 (1.5%)	6 (1,2%)	1.29 (0.51–3.26)
Thrombolytics	16 (1.3%)	0	–
DOACs	0	3 (0.2%)	–
Inferior vena cava filter	23 (1.9%)	7 (1.4%)	1.41 (0.60–3.32)
Long-term therapy			
LMWH	764 (63%)	349 (68%)	0.83 (0.67–1.03)
Mean LMWH dose (IU/kg/day)	156 ± 42	153 ± 45	*p* = 0.28
Vitamin K antagonists	159 (13%)	83 (16%)	0.79 (0.59–1.06)
Fondaparinux	13 (1.1%)	8 (1.5%)	0.69 (0.29–1.68)
DOACs	7 (0.6%)	1 (0.2%)	3.01 (0.37–24.5)

Abbreviations: CI, confidence intervals; DOACs, direct oral anticoagulants; IU, international units; LMWH, low-molecular-weight heparin; SD, standard deviation.

**Fig. 1:**
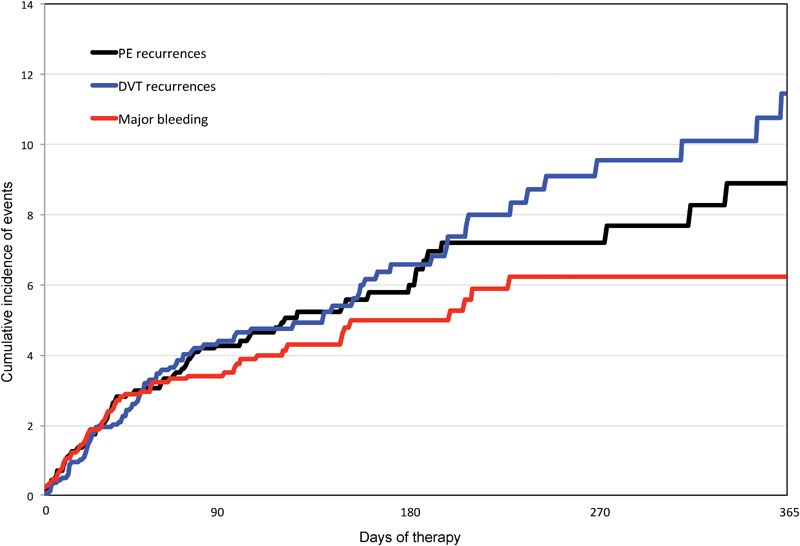
Cumulative rate of PE recurrences, DVT recurrences, and major bleeding during anticoagulation. DVT, deep vein thrombosis; PE, pulmonary embolism.

During the course of anticoagulation (median duration, 93 days), 166 patients developed symptomatic VTE recurrences (recurrent PE: 80, recurrent DVT: 86), 63 had major bleeding (gastrointestinal: 27, intracranial: 11), and 870 died ( Table 3 ). There were no differences in the rates of PE recurrences (HR: 0.76; 95% CI: 0.48–1.20), major bleeding (HR: 0.99; 95% CI: 0.58–1.70), or death (HR: 1.03; 95% CI: 0.89–1.18) between patients with PE and DVT, but patients initially presenting with PE had a lower rate of DVT recurrences (HR: 0.62; 95% CI: 0.40–0.96) than those with DVT. Interestingly, the rate of VTE recurrences was twofold higher than the rate of major bleeding during the first 30 days of anticoagulant therapy (61 vs. 33 events) and over fourfold higher (105 vs. 30 events) beyond the first month ( Table 3 and Fig. 1 ).

**Table 3:** Clinical outcomes during the course of anticoagulant therapy, according to initial VTE presentation

	Pulmonary embolism		Deep vein thrombosis		Hazard ratio (95% CI)
	*N*	Events per 100 patient-years	*N*	Events per 100 patient-years	
**Patients** , ***N***	**1,208**		**517**		
Patient-years of therapy	492.6		175.2		
Recurrent PE	52	10.6 (7.88–13.8)	28	16.0 (10.6–23.1)	0.76 (0.48–1.20)
Recurrent DVT	52	10.6 (7.88–13.8)	34	19.4 (13.4–27.1)	0.62 (0.40–0.96)
Recurrent VTE	104	21.1 (17.3–2.56)	62	35.4 (27.1–45.4)	0.68 (0.50–0.94)
Major bleeding	44	8.93 (6.49–12.0)	19	10.8 (6.53–16.9)	0.99 (0.58–1.70)
Site of major bleeding					
Gastrointestinal	16	3.25 (1.86–5.28)	11	6.28 (3.13–11.2)	0.63 (0.29–1.36)
Cerebral	7	1.42 (0.57–2.93)	4	2.28 (0.61–5.85)	0.73 (0.21–2.51)
Death	622	126 (117–137)	248	142 (125–160)	1.03 (0.89–1.19)
Causes of death					
Initial PE	39	7.92 (5.63–10.8)	–	–	–
Recurrent PE	13	2.64 (1.40–4.51)	5	2.85 (0.92–6.66)	1.15 (0.41–3.22)
Bleeding	9	1.83 (0.83–3.47)	6	3.42 (1.25–7.46)	0.66 (0.23–1.85)
Respiratory insufficiency	49	9.95 (7.36–13.1)	12	6.85 (3.54–12.0)	1.77 (0.94–3.33)
Sudden, unexpected	4	0.81 (0.22–2.08)	2	1.14 (0.13–4.12)	0.89 (0.16–4.85)
30-d outcomes					
Recurrent PE	25 (2.1%)		12 (2.3%)		0.92 (0.46–1.83)
Recurrent DVT	16 (1.3%)		13 (2.5%)		0.54 (0.26–1.13)
Recurrent VTE	41 (3.4%)		25 (4.8%)		0.72 (0.44–1.19)
Major bleeding	24 (2.0%)		12 (2.3%)		0.88 (0.44–1.77)
Death	269 (22%)		92 (18%)		1.29 (1.02–1.63)
Fatal PE	8 (0.7%)		3 (0.6%)		1.18 (0.31–4.44)
Fatal bleeding	6 (0.5%)		5 (1.0%)		0.53 (0.16–1.74)

Abbreviations: CI, confidence intervals; DVT, deep vein thrombosis; PE, pulmonary embolism; VTE, venous thromboembolism.

**Fig. 2:**
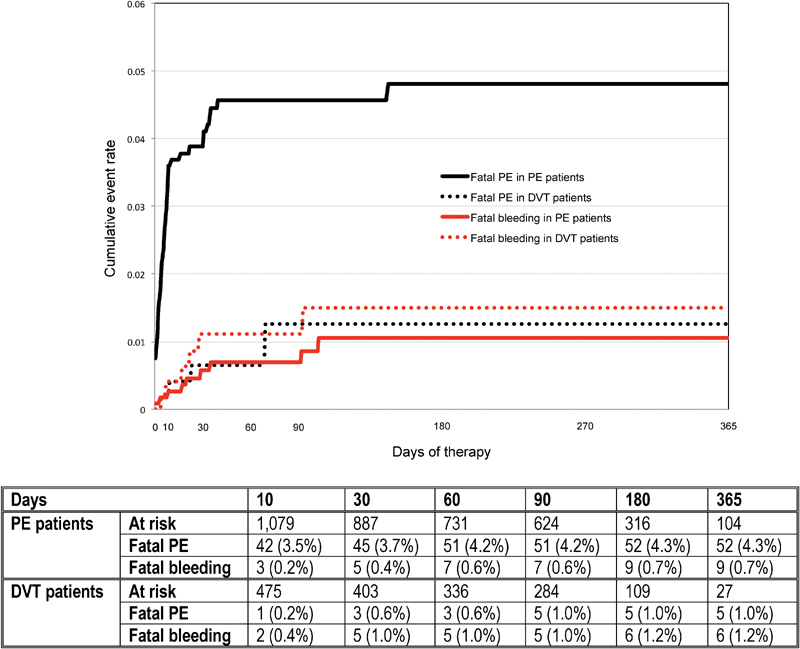
Cumulative rates of fatal PE and fatal bleeding, according to initial VTE presentation. DVT, deep vein thrombosis; PE, pulmonary embolism.

Of 870 patients who died during anticoagulation, 57 (6.6%) died of PE (9 during the first 24 hours) and 15 (1.7%) died of bleeding. Most fatal PEs (48 of 57, 84%) and most fatal bleeds (10 of 15, 67%) occurred within the first 30 days of therapy ( Fig. 2 ). The case-fatality rate of PE recurrences (18 of 80 events were fatal: 22.5%; 95% CI: 14.4–32.6) and major bleeding (15 of 63 bleeds: 23.8%; 95% CI: 14.5–35.5) was similar. Compared with patients who did not die of PE or bleeding, those with fatal PE were more likely to initially present with PE, to be immobilized recently (only 28% had received VTE prophylaxis), to be using corticosteroids, to have raised platelet count at baseline, or to be initially treated with UFH ( Table 4 ). Of 72 patients dying of PE or bleeding, 15 (21%) had no metastases, 26 (36%) were diagnosed with cancer less than 3 months before, and 29 (40%) developed VTE shortly after surgery or immobility.

**Table 4:** Clinical characteristics of patients who died of PE or bleeding

	Fatal PE	Fatal bleeding	Neither
**Patients** , ***N***	**57**	**15**	**1,653**
Clinical characteristics			
Male gender	38 (67%)	12 (80%)	1,200 (73%)
Age <50 y	3 (5.3%)	2 (13%)	168 (10%)
Initial VTE presentation			
Pulmonary embolism	52 (91%) ^‡^	9 (60%)	1,147 (69%)
Cancer characteristics			
Metastases	47 (83%)	10 (67%)	1,213 (73%)
Time since cancer diagnosis			
< 90 d	21 (37%)	5 (33%)	839 (51%)
< 30 d	8 (14%)	1 (6.7%)*	537 (33%)
Cancer therapy			
Radiotherapy	2 (3.5%)	1 (6.7%)	75 (4.5%)
Chemotherapy	28 (49%)	7 (47%)	631 (38%)
Radio- and chemotherapy	7 (12%)	3 (20%)	204 (12%)
Underlying conditions			
Chronic lung disease	19 (33%)	4 (27%)	392 (24%)
Chronic heart failure	6 (11%)	1 (6.7%)	84 (5.1%)
Additional VTE risk factors			
Postoperative	2 (3.5%)	0	127 (7.7%)
Immobility ≥4 d	23 (40%) ^‡^	4 (27%)	319 (19%)
Use of estrogens	1 (18%)	1 (6.7%)*	16 (1.0%)
None of the above	33 (58%) ^†^	10 (67%)	1,219 (74%)
Prior VTE	5 (8.8%)	3 (20%)	181 (11%)
Concomitant therapies			
Antiplatelets	3 (5.3%)	2 (13%)	230 (14%)
Corticosteroids	21 (37%)*	5 (33%)	368 (22%)
Blood tests			
Anemia	33 (58%)	11 (73%)	963 (58%)
Platelet count <100,000/µL	3 (5.3%)	3 (20%)	126 (7.6%)
Platelet count >450,000/µL	10 (18%) ^‡^	2 (13%)	103 (6.2%)
CrCl levels <60 mL/min	18 (32%)	5 (33%)	445 (27%)
Initial therapy			
LMWH	46 (81%)*	14 (93%)	1,485 (90%)
LMWH dose <175 IU/kg/d	13 (23%)*	4 (27%)	589 (36%)
Unfractionated heparin	9 (16%) ^†^	1 (6.7%)	102 (6.2%)
Inferior vena cava filter	1 (1.8%) ^‡^	1 (6.7%)	28 (1.7%)

Abbreviations: CrCl, creatinine clearance; IU, international units; LMWH, low-molecular-weight heparin; PE, pulmonary embolism; VTE, venous thromboembolism.

Notes: Patients with fatal PE or fatal bleeding were compared with patients with neither: * *p* <0.05; ^†^ *p* < 0.01; ^‡^ *p* < 0.001.

On multivariable analyses, patients with CrCl levels less than 60 mL/min were at lower risk for PE recurrences (HR: 0.52; 95% CI: 0.28–0.96) and those with recent major bleeding at baseline were at increased risk for major bleeding during anticoagulation (HR: 4.01; 95% CI: 1.36–11.9). We failed to find other independent predictors for these outcomes.

## Discussion

Our data, obtained from a large series of patients with lung cancer and symptomatic VTE, reveal that 72 of 870 patients (8.3%) dying during the course of anticoagulant therapy died of PE or bleeding. Of these, one in every five patients (21%) had no metastases. Thus, the clinical relevance of VTE in lung cancer patients should not be underestimated. Interestingly, VTE often appeared early in the course of lung cancer: half (50%) of the patients had been diagnosed with cancer less than 3 months prior to VTE, 32% less than 30 days before. One in every three patients (32%) had not received chemo- or radiotherapy yet. Thus, active surveillance on early signs and symptoms of VTE appearing in patients with recently diagnosed lung cancer might likely help detect VTE earlier, and treat it more efficiently. Previous studies also reported that the risk of VTE is highest in the first few months after the diagnosis of several malignancies, including the lung. 2 14 15 16

One in every nine patients (16%) with fatal PE in our cohort died within the first 24 hours, with few time for anticoagulant therapy to be effective. Therefore, accurate identification of at-risk patients would likely help prescribe adequate preventive measures and to detect VTE earlier. In our cohort, one in every four patients (28%) dying of PE or bleeding developed VTE shortly after surgery or immobilization, but only 65 and 32%, respectively, did receive prophylaxis. A wider prescription of effective and safe prophylaxis of VTE (particularly during periods of immobility) might have reduced the amount of patients developing VTE, and thus the amount of patients dying of PE or bleeding.

Over half of the patients (58%) in our cohort had anemia, one in every four had renal insufficiency (27%) or were using corticosteroids (23%), and one in every five (21%) had thrombocytopenia. Not unexpectedly, the rate of major bleeding events appearing during the course of anticoagulant therapy was high (10 major bleeds per 100 patient-years). However, the rate of VTE recurrences was over twofold higher than the rate of major bleeding, and the rate of fatal PE also exceeded the rate of fatal bleeding (57 and 15 deaths, respectively). Since up to half of the VTE recurrences and major bleeds did appear during the first 30 days of therapy, this is the time when patients should be more closely monitored. Unfortunately, however, we failed to find independent predictors for VTE recurrences or major bleeding, other than renal function and recent major bleeding.

As in previous cohort studies and randomized clinical trials, the highest rates of VTE recurrences and major bleeding in our cohort occurred during the first weeks of therapy. 2 17 18 19 20 21 22 23 In a recent study, we reported that the rate of VTE recurrences during anticoagulation was similar to the rate of major bleeding in patients with breast or colorectal cancer, much higher in those with lung cancer, and lower in patients with prostatic cancer. 24 These findings may be explained by differences in the characteristics of the different tumors or their therapies, and suggest that different clinical profiles with regard to VTE-related outcomes are observed according to the primary cancer site. These data support the development of specific research addressed to evaluate cancer-specific anticoagulant strategies with regard to intensity and duration that would help tailoring VTE management.

To our knowledge, ours is the largest cohort of patients with lung cancer and VTE. Our study, however, has several limitations. First, as an observational registry, patients in RIETE were not treated with a standardized regimen; treatment varied with local practices and is likely to have been influenced by a physician's assessment of a patient's risk of bleeding or recurrent events. Factors including type, extent and rate of progression of cancer, concomitant chemotherapy, affordability of treatment, and patient preferences would have all influenced the outcomes. Second, RIETE does not have a central adjudication committee, and the causes of death relied on the reports by attending doctors. Certainly, some doctors may have underestimated the relative frequency of fatal PEs or fatal bleeding, since we looked at symptomatic VTE recurrences and bleeding events, but there could have been several patients dying at home who were considered to die of “cancer,” or of unknown reasons. On the contrary, the risk to have overestimated these risks seems lower given the strict definition of fatal PE and fatal bleeding in RIETE. Finally, the median duration of anticoagulation was only 3 months. This was largely due to the high mortality rate in our cohort, since 50% of patients died during follow-up.

In conclusion, one in every five patients dying of PE or bleeding had no metastases and one in every three had developed the VTE shortly after surgery or immobility. Since half of the patients had been diagnosed with cancer less than 3 months prior to VTE, we suggest that active surveillance on early signs and/or symptoms of VTE in patients with recently diagnosed lung cancer and prescription of VTE prophylaxis in patients undergoing surgery or during periods of immobilization might likely help prevent VTE better, detect it earlier, and treat it more efficiently.

## Notes

Appendix A
**Coordinator of the RIETE Registry:** Manuel Monreal.

**RIETE Steering Committee Members:** Hervé Decousus, Paolo Prandoni, Benjamin Brenner.

**RIETE National Coordinators:** Raquel Barba (Spain); Pierpaolo Di Micco (Italy); Laurent Bertoletti (France); Inna Tzoran (Israel); Abilio Reis (Portugal); Marijan Bosevski (R. Macedonia); Henri Bounameaux (Switzerland); Radovan Malý (Czech Republic); Philip Wells (Canada); Peter Verhamme (Belgium).

**RIETE Registry Coordinating Center:** S&H Medical Science Service.

**Members of the RIETE Group:** SPAIN: Aibar MA, Aibar J, Alfonso M, Amado C, Aranda C, Arcelus JI, Arenas A, Ballaz A, Barba R, Barrón M, Barrón-Andrés B, Bascuñana J, Blanco-Molina A, Bolado C, Camon AM, Carrasco C, Castejón-Pina N, Chasco L, Cruz AJ, de Ancos C, del Pozo R, del Toro J, Díaz-Pedroche MC, Díaz-Peromingo JA, Falgá C, Farfán AI, Fernández-Capitán C, Fidalgo MA, Font C, Font L, Furest I, García MA, García-Bragado F, García-Morillo M, Gavín O, Gil A, Gómez V, Gómez-Cuervo C, González-Martínez J, Grau E, Guijarro R, Guirado L, Gutiérrez J, Hernández-Blasco L, Jara-Palomares L, Jaras MJ, Jiménez D, Jou I, Joya MD, Lima J, Lobo JL, López-Jiménez L, López-Miguel P, López-Nuñez JJ, López-Reyes R, López-Sáez JB, Lorente MA, Lorenzo A, Loring M, Loscos S, Lumbierres M, Maestre A, Marchena PJ, Martínez-Baquerizo C, Martín-Asenjo M, Martín-Fernández M, Martín-Guerra JM, Monreal M, Morales MV, Nieto JA, Núñez MJ, Olivares MC, Otalora S, Otero R, Pedrajas JM, Pellejero G, Pérez-Ductor C, Peris ML, Pesce ML, Porras JA, Riesco D, Rivas A, Rodríguez-Dávila MA, Rodríguez-Galán I, Rodríguez-Hernández A, Rosa V, Rubio CM, Ruiz-Artacho P, Ruiz-Ruiz J, Sahuquillo JC, Sala-Sainz MC, Sampériz A, Sánchez-Muñoz-Torrero JF, Sancho T, Sanoja ID, Soler S, Soto MJ, Suriñach JM, Torres MI, Trujillo-Santos J, Uresandi F, Usandizaga E, Valle R, Vela J, Vilar C, Villalobos A; ARGENTINA: Gutiérrez P, Vázquez FJ, Vilaseca A; BELGIUM: Vanassche T, Vandenbriele C, Verhamme P; BRAZIL: Yoo HHB; CZECH REPUBLIC: Hirmerova J, Malý R; ECUADOR: Salgado E; FRANCE: Benzidia I, Bertoletti L, Bura-Riviere A, Farge-Bancel D, Hij A, Merah A, Mahé I, Moustafa F; ISRAEL: Braester A, Brenner B, Ellis M, Tzoran I; ITALY: Bilora F, Bortoluzzi C, Brandolin B, Bucherini E, Ciammaichella M, Dentali F, Di Micco P, Di Pangrazio M, Grandone E, Imbalzano E, Maida R, Mastroiacovo D, Ngoc V, Pace F, Parisi R, Pesavento R, Prandoni P, Quintavalla R, Rocci A, Siniscalchi C, Sotgiu P, Tiraferri E, Tonello D, Tufano A, Ventresca A, Visonà A; LATVIA: Kalejs RV, Skride A, Vitola B; REPUBLIC OF MACEDONIA: Bosevski M, Zdraveska M; SWITZERLAND: Bounameaux H, Fresa M, Mazzolai L, Ney B, USA: Caprini J; VIETNAM: Bui HM.

## References

[1] Siegel R Naishadham D Jemal A Cancer statistics, 2012 CA Cancer J Clin 2012 62 01 10 29 22237781 10.3322/caac.20138

[2] Chew H K Davies A M Wun T Harvey D Zhou H White R H The incidence of venous thromboembolism among patients with primary lung cancer J Thromb Haemost 2008 6 04 601 608 18208538 10.1111/j.1538-7836.2008.02908.x

[3] Paneesha S McManus A Arya R Frequency, demographics and risk (according to tumour type or site) of cancer-associated thrombosis among patients seen at outpatient DVT clinics Thromb Haemost 2010 103 02 338 343 20024496 10.1160/TH09-06-0397

[4] Horsted F West J Grainge M J Risk of venous thromboembolism in patients with cancer: a systematic review and meta-analysis PLoS Med 2012 9 07 e1001275 22859911 10.1371/journal.pmed.1001275 PMC3409130

[5] Riedl J Posch F Königsbrügge O Red cell distribution width and other red blood cell parameters in patients with cancer: association with risk of venous thromboembolism and mortality PLoS One 2014 9 10 e111440 25347577 10.1371/journal.pone.0111440 PMC4210186

[6] Zhang Y Yang Y Chen W Prevalence and associations of VTE in patients with newly diagnosed lung cancer Chest 2014 146 03 650 658 24676401 10.1378/chest.13-2379

[7] Connolly G C Menapace L Safadjou S Francis C W Khorana A A Prevalence and clinical significance of incidental and clinically suspected venous thromboembolism in lung cancer patients Clin Lung Cancer 2013 14 06 713 718 23891560 10.1016/j.cllc.2013.06.003

[8] Monreal M Kakkar A K Caprini J A The outcome after treatment of venous thromboembolism is different in surgical and acutely ill medical patients. Findings from the RIETE registry J Thromb Haemost 2004 2 11 1892 1898 15550017 10.1111/j.1538-7836.2004.01012.x

[9] Laporte S Mismetti P Décousus H Clinical predictors for fatal pulmonary embolism in 15,520 patients with venous thromboembolism: findings from the Registro Informatizado de la Enfermedad TromboEmbolica venosa (RIETE) Registry Circulation 2008 117 13 1711 1716 18347212 10.1161/CIRCULATIONAHA.107.726232

[10] Muñoz-Torrero J F Bounameaux H Pedrajas J M Effects of age on the risk of dying from pulmonary embolism or bleeding during treatment of deep vein thrombosis J Vasc Surg 2011 54(6, Suppl):26S 32S 10.1016/j.jvs.2011.05.114 21908150

[11] Trujillo-Santos J Schellong S Falga C Low-molecular-weight or unfractionated heparin in venous thromboembolism: the influence of renal function Am J Med 2013 126 05 425 434 23499331 10.1016/j.amjmed.2012.09.021

[12] Farge D Trujillo-Santos J Debourdeau P Fatal events in cancer patients receiving anticoagulant therapy for venous thromboembolism Medicine (Baltimore) 2015 94 32 e1235 26266353 10.1097/MD.0000000000001235 PMC4616675

[13] Bikdeli B Jimenez D Hawkins M Rationale, Design and methodology of the computerized registry of patients with venous thromboembolism (RIETE) Thromb Haemost 2018 118 01 214 224 29304541 10.1160/TH17-07-0511 PMC5821113

[14] Blom J W Doggen C J Osanto S Rosendaal F R Malignancies, prothrombotic mutations, and the risk of venous thrombosis JAMA 2005 293 06 715 722 15701913 10.1001/jama.293.6.715

[15] Blom J W Osanto S Rosendaal F R The risk of a venous thrombotic event in lung cancer patients: higher risk for adenocarcinoma than squamous cell carcinoma J Thromb Haemost 2004 2 10 1760 1765 15456487 10.1111/j.1538-7836.2004.00928.x

[16] Kourelis T V Wysokinska E M Wang Y Yang P Mansfield A S Tafur A J Early venous thromboembolic events are associated with worse prognosis in patients with lung cancer Lung Cancer 2014 86 03 358 362 25453848 10.1016/j.lungcan.2014.10.003 PMC5046820

[17] Chee C E Ashrani A A Marks R S Predictors of venous thromboembolism recurrence and bleeding among active cancer patients: a population-based cohort study Blood 2014 123 25 3972 3978 24782507 10.1182/blood-2014-01-549733 PMC4064333

[18] Francis C W Kessler C M Goldhaber S Z Treatment of venous thromboembolism in cancer patients with dalteparin for up to 12 months: the DALTECAN Study J Thromb Haemost 2015 13 06 1028 1035 25827941 10.1111/jth.12923

[19] Meyer G Marjanovic Z Valcke J Comparison of low-molecular-weight heparin and warfarin for the secondary prevention of venous thromboembolism in patients with cancer: a randomized controlled study Arch Intern Med 2002 162 15 1729 1735 12153376 10.1001/archinte.162.15.1729

[20] Lee A Y Levine M N Baker R I Low-molecular-weight heparin versus a coumarin for the prevention of recurrent venous thromboembolism in patients with cancer N Engl J Med 2003 349 02 146 153 12853587 10.1056/NEJMoa025313

[21] Hull R D Pineo G F Brant R F Long-term low-molecular-weight heparin versus usual care in proximal-vein thrombosis patients with cancer Am J Med 2006 119 12 1062 1072 17145251 10.1016/j.amjmed.2006.02.022

[22] Lee A YY Kamphuisen P W Meyer G Tinzaparin vs warfarin for treatment of acute venous thromboembolism in patients with active cancer: a randomized clinical trial JAMA 2015 314 07 677 686 26284719 10.1001/jama.2015.9243

[23] van Es N Louzada M Carrier M Predicting the risk of recurrent venous thromboembolism in patients with cancer: a prospective cohort study Thromb Res 2018 163 41 46 29353682 10.1016/j.thromres.2018.01.009

[24] Mahé I Chidiac J Bertoletti L The clinical course of venous thromboembolism may differ according to cancer site Am J Med 2017 130 03 337 347 27884650 10.1016/j.amjmed.2016.10.017

